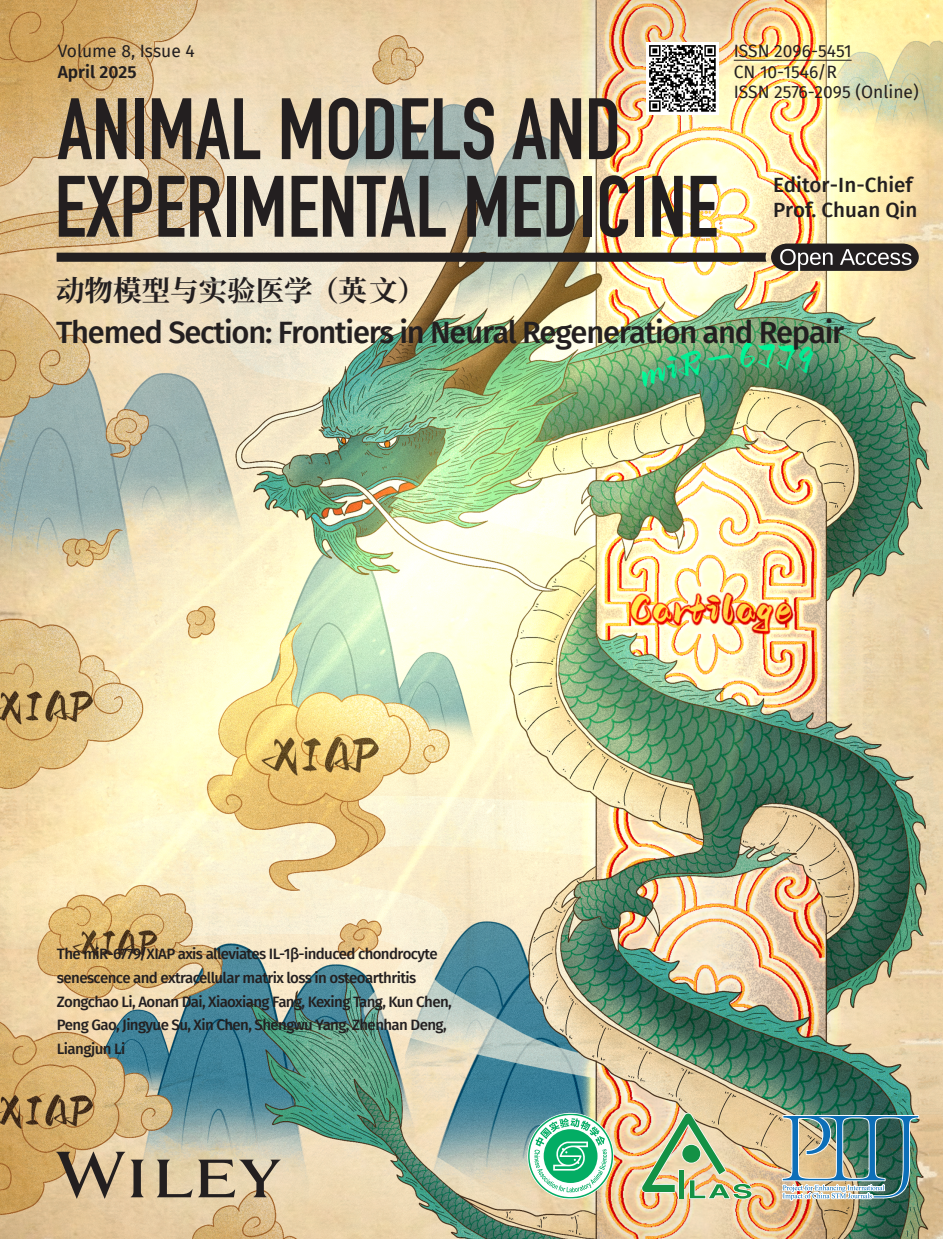# Cover Picture

**DOI:** 10.1002/ame2.12428

**Published:** 2025-04-18

## Abstract

The design draws inspiration from traditional Chinese Taoist culture, where the dragon—one of the Four Celestial Guardians—symbolizes protection, vitality, and the dispelling of negative forces. Abstract Column (Skeletal Structure): the column represents bone, stylized with intricate patterns to evoke cartilage texture, subtly illustrating the joint's anatomical framework. Emerald Dragon (miR‐6779): the dragon, rendered in green to symbolize life force and renewal, embodies miR‐6779. Its elongated form mirrors the molecule's structure while emphasizing its role in mitigating cartilage senescence. Dispelling Mist (XIAP Suppression): swirling mist signifies XIAP, a driver of cartilage degradation in osteoarthritis (OA). The dragon actively disperses the mist, visually capturing the suppression of XIAP expression and the subsequent protection of cartilage integrity. Harmonized Ecosystem: the final scene transitions from fog to clarity (“clearing clouds to reveal sunlight”), metaphorizing the restoration of cartilage homeostasis and the study's therapeutic promise.